# Analysis of circRNA and miRNA expression profiles in IGF3-induced ovarian maturation in spotted scat (*Scatophagus argus*)

**DOI:** 10.3389/fendo.2022.998207

**Published:** 2022-11-25

**Authors:** Zhiyuan Li, Yuwen Guo, Charles Brighton Ndandala, Huadong Chen, Chunren Huang, Guangwen Zhao, Hai Huang, Guangli Li, Huapu Chen

**Affiliations:** ^1^ Guangdong Provincial Key Laboratory of Pathogenic Biology and Epidemiology for Aquatic Economic Animals, Guangdong Research Center on Reproductive Control and Breeding Technology of Indigenous Valuable Fish Species, Fisheries College, Guangdong Ocean University, Zhanjiang, China; ^2^ Southern Marine Science and Engineering Guangdong Laboratory (Zhanjiang), Zhanjiang, China; ^3^ Guangdong Havwii Agriculture Group Co., LTD, Zhanjiang, China; ^4^ Hainan Chenhai Aquatic Co., LTD, Sanya, China; ^5^ Key Laboratory of Utilization and Conservation for Tropical Marine Bioresources of Ministry of Education, Hainan Key Laboratory for Conservation and Utilization of Tropical Marine Fishery Resources, Yazhou Bay Innovation Institute, Hainan Tropical Ocean University, Sanya, China

**Keywords:** IGF3, oocyte maturation, ovary, transcriptome, circRNA, miRNA, *scatophagus argus*

## Abstract

Insulin-like growth factor 3 (IGF3) induces ovarian maturation in teleosts; however, research on its molecular regulatory mechanism remains deficient. Circular RNAs (circRNAs) and microRNAs (miRNAs) are involved in various biological processes, including reproduction. In this study, circRNAs and miRNAs involved in IGF3-induced ovarian maturation were evaluated in spotted scat (*Scatophagus argus*). In ovarian tissues, we identified 176 differentially expressed (DE) circRNAs and 52 DE miRNAs between IGF3 treatment and control groups. Gene Ontology (GO) enrichment analyses showed that host genes of DE circRNAs and target genes of DE miRNAs were enriched for various processes with a high degree of overlap, including cellular process, reproduction, reproductive process, biological adhesion, growth, extracellular region, cell junction, catalytic activity, and transcription factor activity. Enriched Kyoto Encyclopedia of Genes and Genomes (KEGG) pathways included cell adhesion molecules, ECM–receptor interaction, regulation of actin cytoskeleton, focal adhesion, cell cycle, Hedgehog signaling pathway, phosphatidylinositol signaling system, PI3K-Akt signaling pathway, Apelin signaling pathway, Notch signaling pathway, insulin signaling pathway, and Rap1 signaling pathway. A circRNA–miRNA–mRNA regulatory network was constructed, including DE genes involved in reproduction (e.g., oocyte maturation, oocyte meiosis, and ECM remodeling), such as *ccnd2*, *hecw2*, *dnm2*, *irs1*, *adam12*, and *cdh13*. According to the regulatory network and tissue distribution, we identified one circRNA (Lachesis_group5:6245955|6270787) and three miRNAs (novel_miR_622, novel_miR_980, and novel_miR_64) that may exert regulatory effects in IGF3-induced ovarian maturation in *S. argus*. Taken together, this study provides a novel insight into the molecular mechanisms by which IGF3 functions in ovaries and highlights the effects of circRNAs and miRNAs in reproduction in *S. argus*.

## Introduction

MicroRNAs (miRNAs) are endogenous non-coding RNAs (ncRNAs) with lengths of about 22 nucleotides. They exert post-transcriptional regulatory functions in plants and animals (1). They pair with the 3′ untranslated region (UTR) of the target genes, resulting in mRNA cleavage or translational repression (2; 1). The relationships between miRNAs and mRNAs are complex, e.g., a single miRNA may target several genes, while a single gene may be regulated by multiple miRNAs (3). MiRNAs are critical in the regulation of multiple biological processes, such as cell proliferation, differentiation, apoptosis, and gametogenesis (4–6). There is evidence for the roles of miRNAs in reproduction. For instance, miR-143 is involved in endocrine system regulation in mammalian gonads and functions in the maintenance of pregnancies (7, 8). In *Oreochromis niloticus*, miR-143 is highly expressed in mature gonads, indicating its functions in fish reproduction (9). In *Danio rerio*, the injection of miR430a-*sox9a* increases the proportion of spermatogonia, and the coinjection of miR430a-*sox9a* and miR218a-*sox9b* stimulates the renewal of ovarian follicles (10).

Circular RNAs (circRNAs) are another type of ncRNA. Because their 3′- and 5′-termini are joined together, circRNAs are characterized by covalent closed-loop structures (11). Studies have shown that circRNAs exert regulatory effects on multiple physiological processes (12). Some circRNAs involved in reproductive processes have been identified (13–15). CircRNAs can function as competing endogenous RNAs to regulate expression levels of target genes (16). For instance, circHIPK3 sponges miR-124a to regulate growth in human cells (17), and the circRNA chi_circ_0008219 also acts as a sponge for multiple miRNAs in pre-ovulatory ovarian follicles of goats (18).

The insulin-like growth factor (IGF) system has vital roles in various cellular processes, such as survival, growth, proliferation, and differentiation (19). IGF1 and IGF2 are regarded as typical ligands of the IGF family and are involved in follicle growth, meiotic resumption, and oocyte maturation (20–22). IGF3 is a newly discovered member of the IGF family exclusively found in teleosts (23). It can stimulate ovarian development and maturation in teleosts (24–27). Furthermore, the knockdown of *igf3* in *Cyprinus carpio via* RNA interference significantly altered ncRNA expression profiles (28), indicating that ncRNAs are involved in IGF3-induced oocyte maturation; however, the molecular regulatory mechanism has not been determined.

Spotted scat (*Scatophagus argus*) is an economically important aquaculture species in south China (29–31) owing to its high nutritional value and colorful appearance (32–34). However, the development of artificial breeding methods for *S. argus* is limited by the inability of the ovaries of cultured *S. argus* to spontaneously mature (35, 36). Considering that IGF3 stimulates ovarian development and maturation in fish and these effects are clearly mediated by ncRNAs, studies of the molecular mechanism underlying IGF3-induced oocyte maturation in *S. argus* are warranted. The purpose of this study was to gain insight into circRNA–miRNA–mRNA interactions involved in the effects of IGF3 on oocyte maturation in *S. argus*.

## Materials and methods

### Experimental treatment and ethics statement

The experimental methods are described in a previous study (25). In brief, the open reading frame of *igf3* in *S. argus* was cloned. The mature peptide of IGF3 was predicted, and the corresponding *igf3* cDNA fragment was amplified. After purification, the *igf3* cDNA fragment was ligated into the pMBP vector and was transfected into the *Escherichia coli* rosetta2 strain to produce recombinant IGF3 protein. After the recombinant IGF3 protein was obtained, *in vitro* experiments were conducted. Three adult female fishes (body weight ranging from 300 to 350 g) were sacrificed, and their ovaries (stage III) were dissected for further experiment. Ovary fragments were washed with Leibovitz’s L-15 medium (Gibco, American), then were incubated in a 24-well culture plate with L-15 medium containing penicillin and streptomycin (100 U/ml, Life Company, Shanghai, China). After pre-incubation (5% CO_2_, 27°C, 2 h), the medium was replaced with fresh medium containing 5 nM recombinant IGF3 protein, and the control group was not supplemented with recombinant IGF3. After incubation for 6 h, ovary fragments were collected and frozen in liquid nitrogen immediately. Then, samples were stored at −80°C for subsequent RNA extraction. The three treated ovary samples and three control ovary samples were used for RNA-seq and smallRNA-seq. In addition, 12 tissues of *S. argus*, including the brain, pituitary, heart, gill, liver, spleen, kidney, stomach, intestines, muscle, ovary, and testis, were collected in triplicate and stored at −80°C for subsequent RNA extraction. The 12 tissue samples were used for tissue distribution analysis of candidate RNAs.

All experimental procedures were in compliance with the Animal Research and Ethics Committees of Fisheries College of Guangdong Ocean University, China.

### RNA library construction and sequencing

RNA extraction was performed with TRIzol (Invitrogen, USA) following the manufacturer’s protocol. The quality and concentration detection of RNA samples were evaluated using the NanoDrop ND-2000 spectrophotometer (NanoDrop Technologies, USA). The purity and integrity of RNA samples were verified by Qubit 2.0 (Thermo Fisher Scientific, USA), Agilent 2100 (Agilent Technologies, USA), and electrophoresis methods. The total RNA with RNA integrity number (RIN) score > 7 was used for sequencing.

After verification of the RNA samples, circRNA libraries were constructed using Ribo-off rRNA Depletion Kit, and miRNA libraries were constructed using VAHTSTM Small RNA Library Prep Kit for Illumina. First rRNA of the samples were removed using the Epicenter RiboZero Kit. For circRNA libraries, linear RNAs were digested using RNase R, and RNA was randomly fragmented using a fragmentation buffer. After synthesis and purification, the cDNA was subjected to end repair, A-tailing, and sequencing adaptor ligation. For miRNA libraries, the small RNAs were ligated to 5′ and 3′ RNA/DNA chimeric oligonucleotide adaptors (Illumina); then, the ligation products were purified, and cDNA was synthesized. After purification and quality control, the cDNA libraries were sequenced on the Illumina NovaSeq 6000 platform (San Diego, USA) using NovaSeq 6000 S4 Reagent Kit by BioMarker Technologies company (Beijing, China). Raw data were uploaded to the National Center for Biotechnology Information (NCBI) (accession number PRJNA849653).

### Sequencing quality control and analysis

To obtain clean data, raw sequencing data were filtered, including the removal of adapters, low-quality reads (*q*-value ≤ 20), or reads containing more than 5% unknown nucleotides (N) (37). Then, clean data were aligned to the genome of *S. argus* (38). The clean data of circRNA were aligned to the genome using HISAT2 with parameter –rna-strandness RF, and the clean data of miRNA were aligned to the genome using Bowtie with parameter -v 0. The mapped data were used for subsequent analyses.

### Prediction and identification of circRNAs and miRNAs

The mapped reads from circRNA libraries were used for circRNA prediction using find_circ with default parameter (39). The mapped reads from miRNA libraries were aligned to the sequences of the mature miRNA in miRBase (v22) to identity the known miRNAs. Then, the novel miRNA were predicted using miRDeep2 with parameter -g -1 -b 0 (39).

### Differential expression analysis

Differentially expressed (DE) circRNAs and miRNAs were detected between the treated group and the control group using DESeq2 with default parameter. The criteria for DE circRNA were fold change (FC) ≥ 2 and *p* < 0.05. The criteria for DE miRNA were fold change (FC) ≥ 1.5 and *p* ≤ 0.01. To avoid false positives, the Benjamini–Hochberg correction method was used, and the FDR was used as the key indicator for the DE miRNAs.

### Functional enrichment analysis

A functional enrichment analysis of host genes of DE circRNAs was conducted with Gene Ontology (GO) and Kyoto Encyclopedia of Genes and Genomes (KEGG) databases using DAVID and KOBAS after non-redundant protein sequence database (NR) annotation. Target genes of miRNAs were predicted using miRanda and TargetScan and were also evaluated by GO and KEGG pathway enrichment analyses after NR annotation. The significance of enrichment analysis was indicated by *p* < 0.05.

### circRNA–miRNA–mRNA network construction

To construct a preliminary network of circRNAs, miRNAs, and mRNAs, we focused on the DE circRNAs and DE miRNAs that related to DE genes with known roles in reproduction and ovarian maturation. According to the predicted circRNA–miRNA and miRNA–mRNA pairs, a circRNA–miRNA–mRNA coexpression network was constructed. The network was visualized using Cytoscape (http://www.cytoscape.org/).

### Validation by real-time quantitative PCR

The cDNA was synthesized using TransScript^®^ Uni All-in-One First-Strand cDNA Synthesis SuperMix for qPCR (One-Step gDNA Removal) (TransGen Biotech, China) The cDNA was then used for RT-qPCR with PerfectStart^®^ Green qPCR SuperMix (TransGen Biotech, China). The conditions were initial denaturation at 95°C for 2 min, followed by 45 cycles of 95°C for 20 s, 55°C for 30 s, and 72°C for 30 s. Relative expressions were calculated by the 2^−ΔΔCt^ method. *β-Actin* was chosen as a reference gene for circRNAs, and *u6* was the reference for miRNAs. All primer sequences are shown in **Table 1**.

**Table 1:** Primer sequences of circRNAs and miRNAs for the qPCR analysis.

Transcript ID	Type	Primers (5′–3′)
Lachesis_group5:6245955|6270787	circRNA	F: GAGGAAAGAAGCCACTGTCG
		R: AGGCCTAACGAGCTCACAAA
Lachesis_group19:16809749|16823164	circRNA	F: CACTGGAAATCTCCCTCGAA
		R: CCAGGTGATAGGTTCCTCCA
Lachesis_group7:7215287|7239736	circRNA	F: GCTTCCATGTGGATGGAGAT
		R: GCAAGGACCTCTTCGATCTG
Lachesis_group9:19406851|19442214	circRNA	F: GACGTTGTGGGCGACTAAAT
		R: AGATCAGCCAGTGCGATTCT
*β-actin*		F: GAGAGGTTCCGTTGCCCAGAG
		R: CAGACAGCACAGTGTTGGCGT
*ssa-miR-143-3p*	miRNA	F: TGAGATGAAGCACTGTAGCTC
*ccr-miR-1*	miRNA	F: TGGAATGTAAAGAAGTATGTAT
novel_miR_696	miRNA	F: TTTGTGATTGGTCGGATGC
novel_miR_594	miRNA	F: TTTTGCAGGGCTCTGGCAGG
novel_miR_622	miRNA	F: TCTCAGAGCTGTGGCTGCT
novel_miR_980	miRNA	F: TCTAGCACGCGGAGGTCT
novel_miR_64	miRNA	F: CTGGATCCTGAGCCCTCTAT
novel_miR_743	miRNA	F: AAGCTGCTGCCCCTGCAA
common R primer for miRNA	miRNA	R: GATCGCCCTTCTACGTCGTAT
*u6*	miRNA	F: CTCGCTTCGGCAGCACA
		R: AACGCTTCACGAATTTGCGT

### Tissue distribution of candidate circRNA and miRNA by RT-qPCR

One circRNA (Lachesis_group5:6245955|6270787) and three miRNAs (novel_miR_622, novel_miR_980, and novel_miR_64) were selected for analyses of expression levels in 12 tissues (brain, pituitary, heart, gill, liver, spleen, kidney, stomach, intestines, muscle, ovary, and testis); these RNAs were associated with the DE genes involved in oocyte maturation. Triplicate total RNA samples from different tissues of adult *S. argus* individuals were prepared and reverse transcribed into cDNA. The methods for RNA isolation, reverse transcription, and RT-qPCR were the same as described above. The primers for circRNAs and miRNAs are listed in **Table 1**.

## Results

### Overview of RNA-Seq

**Figure 1:**
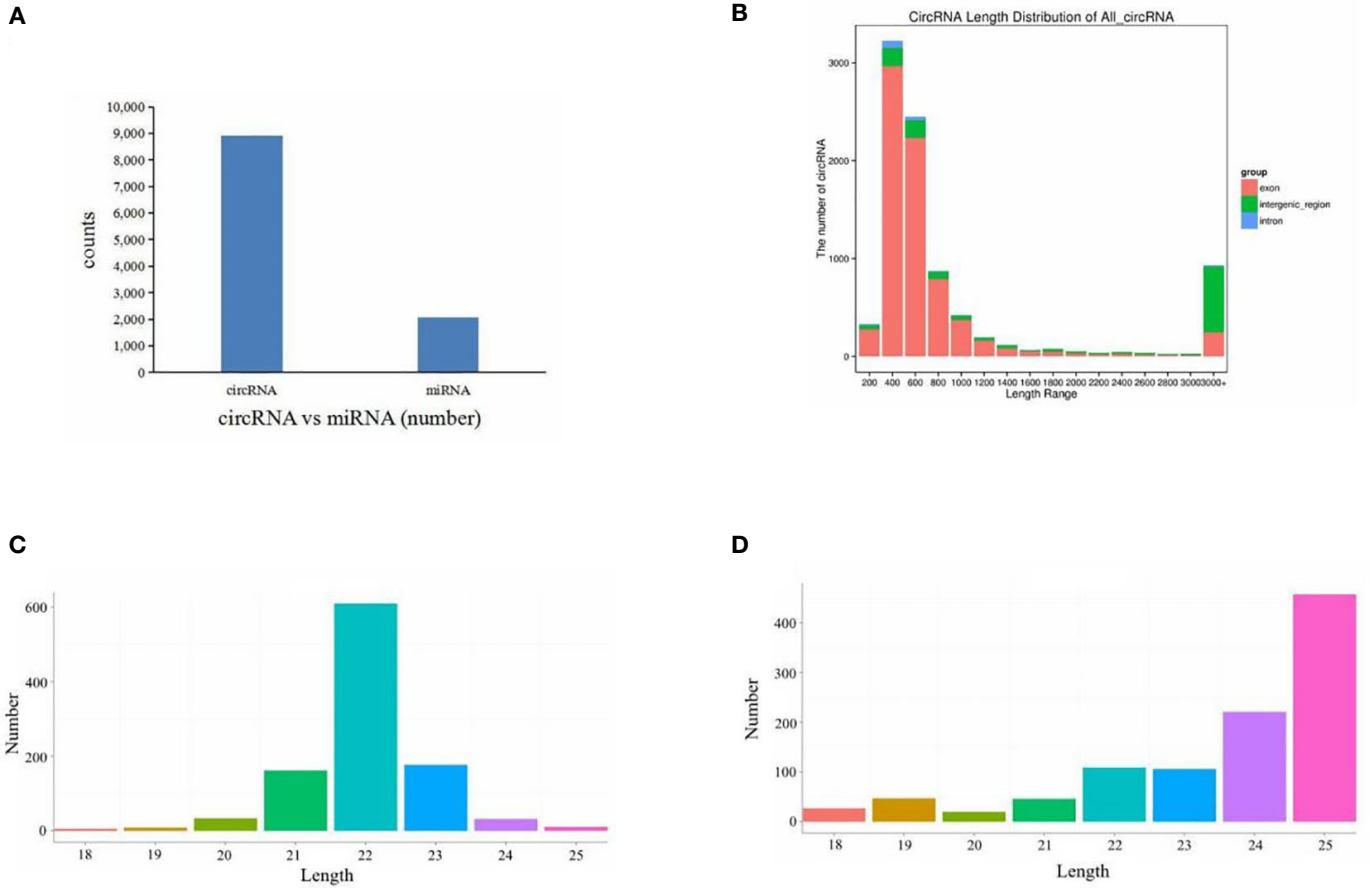
Overview of RNA sequencing. **(A)** Numbers of circRNAs and miRNAs. **(B)** All circRNA length distribution. **(C)** Known miRNA length distribution. **(D)** Novel miRNA length distribution.

Six cDNA circRNA libraries and six cDNA small RNA libraries were constructed and sequenced. After quality control, 102.19 Gb of clean data for circRNA libraries was obtained with an average Q30 above 98.39%, and 2.6 Gb of clean data for small RNA libraries was obtained with an average Q30 above 96.40%. Then, clean reads were aligned to the *S. argus* reference genome. Percentages of clean circRNA reads aligned to the genome ranged from 82.15% to 99.11% (**Table 2**), and percentages of clean small RNA reads aligned to the genome ranged from 81.21% to 86.97% (**Table 3**). The mapped reads were used for further analyses. In total, 8,919 circRNAs and 2,073 miRNAs were identified (**Figure 1**). Among the identified miRNAs, there were 1,040 known miRNAs and 1,033 novel miRNAs.

**Table 2:** Summary of the reads of circRNA libraries after quality control.

Sample	Clean read bases	Q30 (%)	Total clean reads	Mapped reads	Mapping ratio (%)
C 1	14,675,853,774	98.73	97,971,340	97,102,568	99.11
C 2	15,334,296,828	98.64	102,356,164	100,568,020	98.25
C 3	16,207,599,014	98.79	108,158,558	93,843,584	86.76
T 1	18,005,852,362	98.61	120,165,776	98,714,176	82.15
T 2	18,905,444,212	98.39	126,273,552	123,660,736	97.93
T 3	19,062,802,432	98.72	127,205,084	107,092,756	84.19

C refers to the control group; T refers to the IGF3-treated group.

**Table 3:** Summary of the reads of miRNA libraries after quality control.

Sample	Clean read bases	Q30 (%)	Total clean reads	Mapped reads	Mapping ratio (%)
C 1	633,122,894	97.40	23,436,996	20,383,245	86.97
C 2	379,389,279	97.39	14,071,752	12,235,517	86.95
C 3	392,758,093	96.40	14,331,052	11,638,388	81.21
T 1	394,826,818	97.45	14,589,064	12,492,571	85.63
T 2	403,686,383	96.88	14,962,945	12,943,078	86.50
T 3	397,993,450	97.33	14,672,736	12,760,636	86.97

C refers to the control group; T refers to the IGF3-treated group.

### Identification of differentially expressed circRNAs and miRNAs

**Figure 2:**
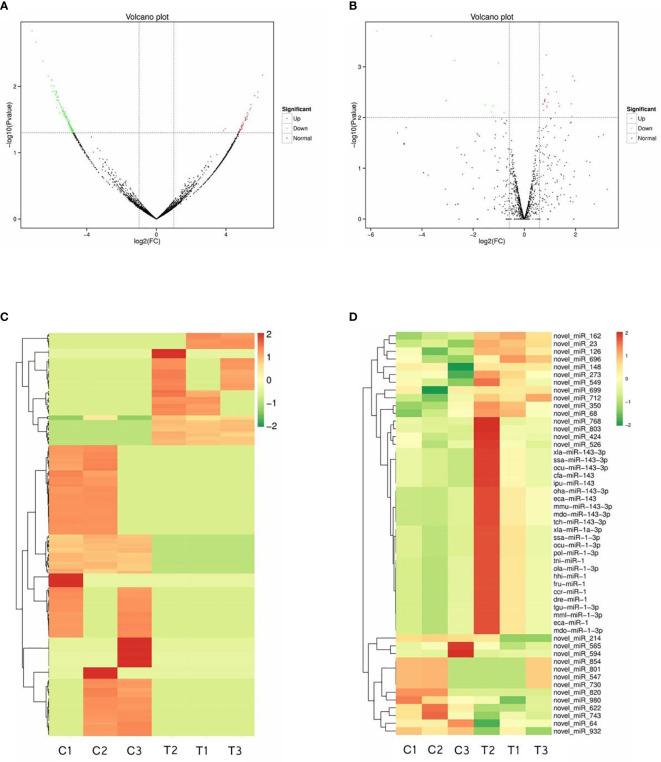
Differentially expressed circRNAs and miRNAs. Volcano plot figures of identified circRNAs **(A)** and miRNAs **(B)**. Heatmaps of differentially expressed circRNAs **(C)** and miRNAs **(D)**.

DE RNAs were discovered by comparisons with the control group. After IGF3 protein treatment, 176 circRNAs and 52 miRNAs were DE. These included 49 upregulated circRNAs and 127 downregulated circRNAs in the treatment group (*p* < 0.05, fold change ≥ 2) and 39 upregulated miRNAs and 13 downregulated miRNAs (*p* < 0.01, fold change ≥ 1.5) (**Supplementary Table S1**). The DE circRNAs and miRNAs are summarized in a volcano plot exhibiting their upregulation or downregulation (**Figures 2A, B**). Results of a systematic clustering analysis of DE circRNAs and miRNAs are shown in heatmaps (**Figures 2C, D**).

### GO and KEGG pathway enrichment analysis

**Figure 3:**
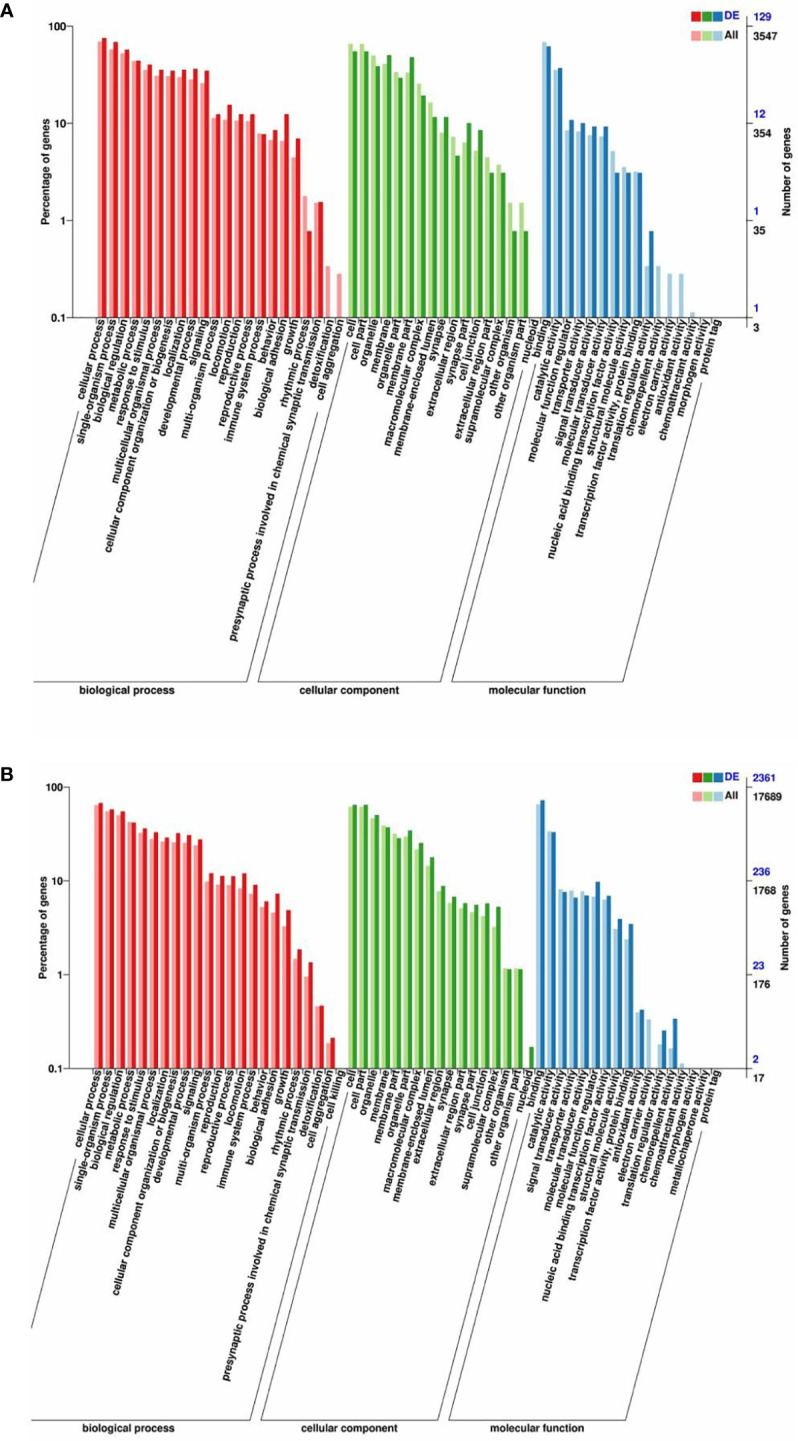
GO enrichment analysis of the host genes of DE circRNAs and the target genes of DE miRNAs. GO enrichment of the host genes of DE circRNAs **(A)** and the target genes of DE miRNAs **(B)**. The terms representing biological process, cellular component, and molecular function are in red, green, and blue, respectively.

Host genes of all DE circRNAs and target genes of all DE miRNAs were predicted and annotated (**Supplementary Table S2**). The top significantly enriched GO terms in three main categories, namely, biological processes, cellular components, and molecular functions, of DE circRNAs and DE miRNAs were determined (**Figures 3A, B**). The enriched GO term for DE circRNAs and DE miRNAs overlapped substantially. In the biological process category, DE circRNAs and DE miRNAs were related to the following terms: cellular process, reproduction, reproductive process, biological adhesion, and growth. In the cellular component category, the terms membrane, extracellular region, cell junction, and extracellular region part were obtained. In the molecular function category, enrichment for binding, catalytic activity, molecular function regulator, nucleic acid binding transcription factor activity, transcription factor activity, and protein binding was detected (**Supplementary Table S3**).

**Figure 4:**
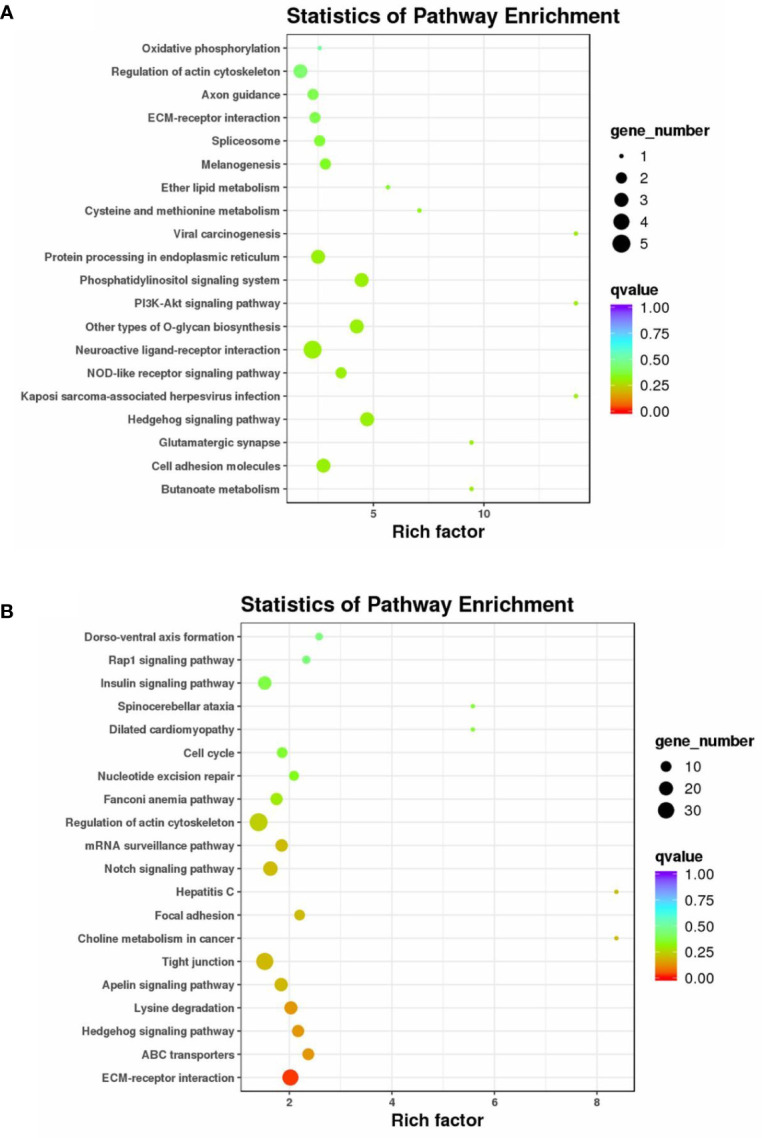
KEGG pathway enrichment analysis of the host genes of DE circRNAs and the target genes of DE miRNAs. KEGG enrichment of the host genes of DE circRNA **(A)** and the target genes of DE miRNAs **(B)**. The color of the dot represents the *q*-value for each pathway. The size of the dot represents the number of genes enriched in each pathway.

A KEGG pathway enrichment analysis was also carried out to uncover the functional pathways defined by DE circRNAs and DE miRNAs (**Supplementary Table S4**). Enriched KEGG pathways involving host genes of DE circRNAs included the Hedgehog signaling pathway, phosphatidylinositol signaling system, PI3K-Akt signaling pathway, cell adhesion molecules, ECM–receptor interaction, and regulation of actin cytoskeleton (**Figure 4A**). Enriched KEGG pathways for target genes of DE miRNAs included ECM–receptor interaction, ABC transporters, Hedgehog signaling pathway, Apelin signaling pathway, tight junction, focal adhesion, Notch signaling pathway, regulation of actin cytoskeleton, cell cycle, insulin signaling pathway, and Rap1 signaling pathway (**Figure 4B**).

### Construction of a circRNA–miRNA–mRNA regulatory network

**Figure 5:**
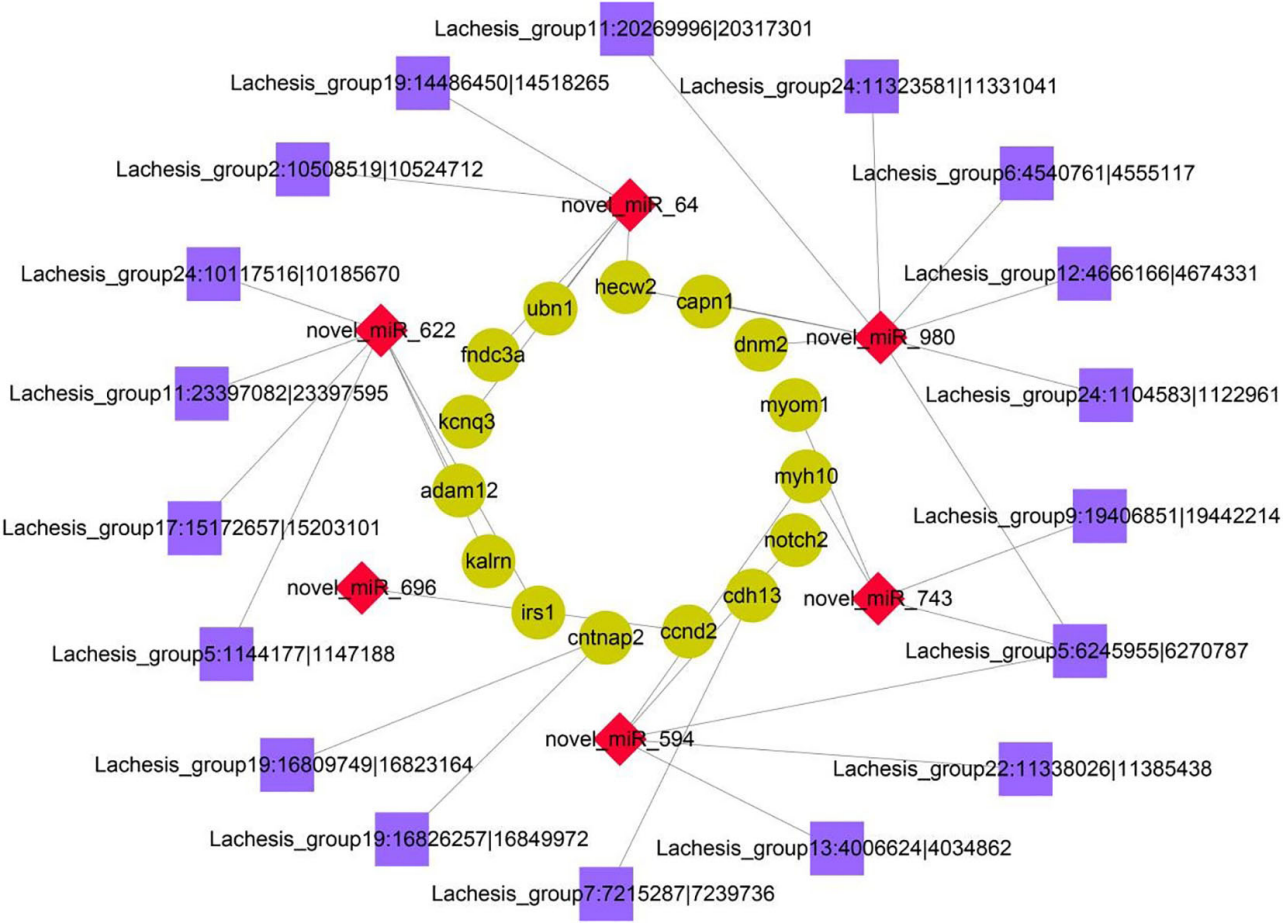
Coexpression network of DE circRNAs, miRNAs, and mRNAs. The cyan circular nodes represent mRNAs, the red diamond nodes represent miRNAs, and the purple square nodes represent circRNAs.

To further reveal the potential regulatory relationship between circRNAs, miRNAs, and mRNAs in IGF3-induced ovarian maturation, the DE mRNAs related to reproduction and ovarian maturation, DE miRNAs targeting these DE mRNAs, and DE circRNAs targeted by these DE miRNAs or related to these DE mRNAs were selected for network construction (**Figure 5**). The network included 18 DE circRNAs, 6 DE miRNAs, and 15 DE mRNAs. In this network, we found three miRNAs (novel_miR_622, novel_miR_980, and novel_miR_64) with multiple connections to DE mRNAs and one circRNA (Lachesis_group5:6245955|6270787) with multiple connections to DE miRNAs.

### Validation by RT-qPCR

**Figure 6:**
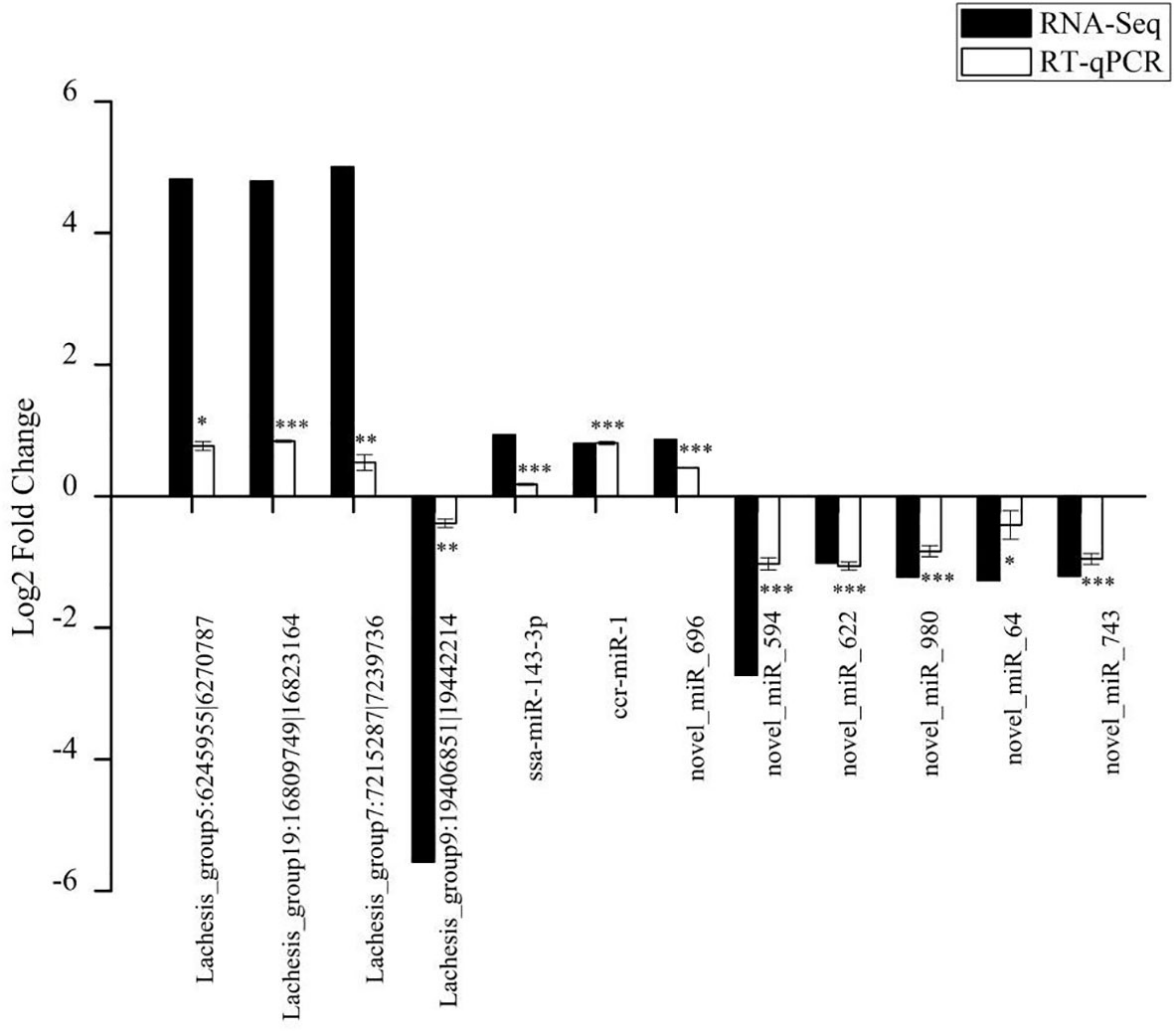
Validation of randomly selected four DE circRNAs and eight DE miRNAs of high-throughput sequencing results using qRT-PCR analysis. Data of qRT-PCR are expressed as the mean value ± SEM (n = 3). *p < 0.05, **p < 0.01, ***p < 0.001.

The RNA-seq results were validated by an RT-qPCR analysis of randomly selected DE circRNAs and miRNAs. The expression trends for all four DE circRNAs and eight DE miRNAs were consistent with the RNA-Seq results (**Figure 6**). The circRNAs (Lachesis_group5:6245955|6270787, Lachesis_group19:16809749|16823164, and Lachesis_group7:7215287|7239736) were upregulated, and the circRNA (Lachesis_group9:19406851|19442214) was downregulated. The miRNAs (ssa-miR-143-3p, ccr-miR-1, and novel_miR_696) were upregulated, and the miRNAs (novel_miR_594, novel_miR_622, novel_miR_980, novel_miR_64, and novel_miR_743) were downregulated. The expression trends for DE mRNAs were also consistent with RT-qPCR results from a previous study (40). Generally, the results of the qPCR analysis validated the RNA-seq results, confirming the accuracy and reliability of the results.

### Tissue distribution of four candidate RNAs

**Figure 7:**
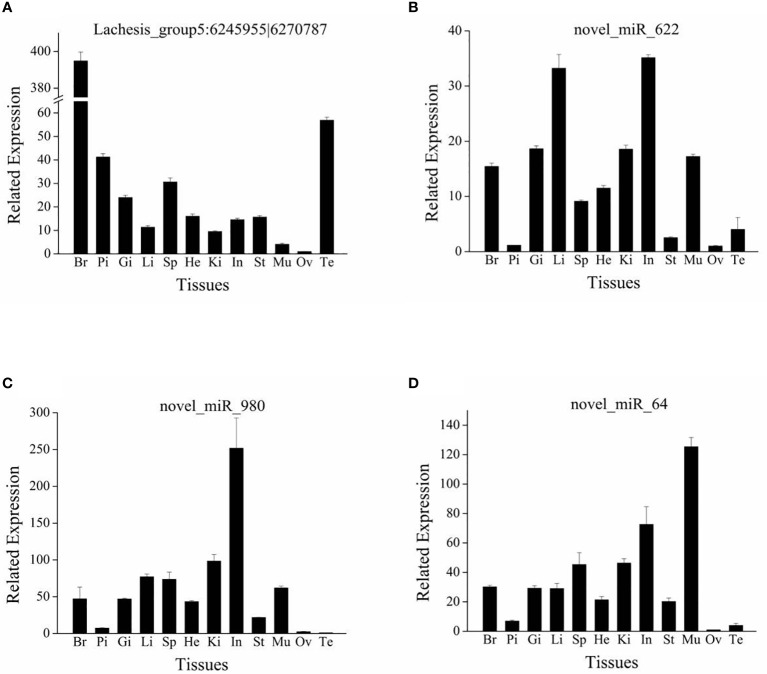
Tissue distribution of circRNA Lachesis_group5:6245955|6270787 **(A)** and three miRNAs novel_miR_622 **(B)**, novel_miR_980 **(C)**, and novel_miR_64 **(D)**. Br, brain; Pi, pituitary; Gi, gill; Li, liver; Sp, spleen; He, heart; Ki, kidney; In, intestine; St, stomach; Mu, muscle; Ov, ovary; Te, testis. Data are expressed as the mean value ± SEM (n = 3).

From the circRNA–miRNA–mRNA regulatory network, we found one circRNA (Lachesis_group5:6245955|6270787) and three miRNAs (novel_miR_622, novel_miR_980, and novel_miR_64) with multiple connections. These RNAs were selected for an analysis of expression distribution in 12 tissues of *S. argus* by RT-qPCR. The four candidate RNAs were all widely distributed across the 12 tissues (**Figure 7**). The circRNA (Lachesis_group5: 6245955|6270787) was expressed differentially in the gonads of different sexes, with low expression in the ovary but high expression in the testis (**Figure 7A**). All three miRNAs were expressed at low levels in gonads, and the expression levels of novel_miR_622 and novel_miR_64 were lowest in the ovary (**Figures 7B–D**).

## Discussion

Oocyte development and maturation, essential processes for successful reproduction, play critical roles in artificial breeding in the aquaculture industry (41). Research has revealed that IGF3 can stimulate oocyte development and maturation in teleosts (24–27). The molecular regulatory mechanism underlying IGF3-induced oocyte maturation remains unclear; accordingly, a RNA-seq analysis was performed in this study. Considering the key roles of miRNAs and circRNAs in reproduction (9, 10, 14), detailed RNA profiles of IGF3-induced oocyte maturation in *S. argus* were obtained. Finally, we identified 176 DE circRNAs and 52 DE miRNAs between the control group and IGF3-treated group.

A KEGG enrichment analysis of DE circRNAs and miRNAs revealed some overlapping terms, such as ECM–receptor interaction, and regulation of actin cytoskeleton. The extracellular matrix (ECM) has contributed to ovarian remodeling during the reproductive cycle and is essential for follicular development and ovulation (42). Additionally, the cytoskeleton plays vital roles in oocyte meiotic maturation by regulating spindle assembly, spindle length, and chromosome segregation (43). Some of the pathways identified in the KEGG enrichment analysis have been the focus of recent research, such as the Hedgehog, PI3K-Akt, Apelin, Notch, insulin, and Rap1 signaling pathways. The hedgehog signaling pathway participates in steroidogenesis in gonad tissues (44, 45), and IGF3 stimulates steroidogenic enzymes in the ovaries of *O. niloticus* (46). In *D. rerio*, IGF3 can induce the maturation of oocytes by activating the PI3K-Akt signaling pathway (23). Apelin13 significantly upregulates the phosphorylation levels of both Akt and AMPK, resulting in the proliferation and migration of cells (47). The Notch signaling pathway is involved in the regulation of female germ cell meiosis progression and early oogenesis (48). Furthermore, this pathway is required for ovarian follicle formation and follicular growth (49). The insulin signaling pathway plays an important role *via* downstream dietary and neuronal signaling in the regulation of various metabolic outputs, including reproductive capacity (50, 51). Furthermore, defects in insulin/IGF1 signaling can cause infertility (52, 53). Rap1b mediates the mitogenic signal of cAMP leading to an increase in G1/S phase entry (54). The activation of Rap1 can activate B-Raf/MEK/Erk and Akt signaling pathways to stimulate cell proliferation and survival and can induce integrin-mediated cell adhesion (55).

In the circRNA–miRNA–mRNA network, several DE genes related to reproduction and ovarian maturation were included. For instance, various DE genes, such as *ccnd2*, *hecw2*, and *dnm2*, were related to oocyte meiotic division, which is a prerequisite for oocyte maturation (56, 57). D-Type cyclins (CCNDs) are cell cycle proteins; CCND2 plays an essential role in oocyte meiosis during the reproductive cycle (58, 59). Furthermore, CCND2 is associated with the proliferation of granulosa cells and ovulation (60). E3 ubiquitin-protein ligase (HECW2) enhances the activation of transcription and is involved in the regulation of the mitotic metaphase/anaphase transition (61, 62). Dynamin 2 (DNM2) binds to and hydrolyzes GTP to produce microtubule bundles and is involved in cytokinesis (63).

During the reproductive cycle, the ECM plays an essential role in ovarian remodeling in follicular development and ovulation (42). Research has revealed that adhesion proteins, such as fibronectin and laminin, are components of the ECM in follicular cells (64, 65). In the network, several cell-adhesion-related genes were detected, such as *adam12* and *cdh13*. The gene *adam12* encodes a member of a family of proteins that are structurally associated with snake venom disintegrins and has been implicated in cell–cell and cell–matrix interactions, such as fertilization (66, 67). Cadherin 13, encoded by *cdh13*, is a member of calcium-dependent cell adhesion proteins and is localized to the surface of the cell membrane (68).

Insulin exerts various effects by binding to the insulin receptor (IR). After ligand binding, the IR undergoes conformational changes to activate various cellular substrates, including the insulin receptor substrate (IRS) (69). Four IRSs have been identified in various mammals (69). All four IRSs are able to interact with all the regulatory subunits of PI3K and to activate the catalytic subunit (70). In the present study, *irs1* was upregulated after IGF3 treatment and was involved in the circRNA–miRNA–mRNA network. IRS1 is reported to be involved in insulin responses, notably mitogenesis (71). A lack of IRS1 in mice leads to defective reproductive function (72).

Among the DE miRNAs, most of the known miRNAs belonged to the miR-143 and miR-1 families. Research has revealed that miR-143 plays an important role in reproduction. At early time points, miR-143 exerts regulatory functions in the formation of primordial follicles, and its regulatory effects continue throughout ovarian development (73). Additionally, miR-143 is highly expressed in mature gonads of *O. niloticus* and *Trachinotus ovatus*, indicating that it regulates fish reproduction during the maturation period (9, 74). Furthermore, miR-143 has been regarded as a critical regulator of the endocrine system in gonadal functions and pregnancy maintenance in mammal (7, 8). It can promote progesterone production in ovarian granulosa cells by interacting with FSH (75). Furthermore, miR-143 is a transcriptional target of the Notch signaling pathway (76) and modulates cytoskeletal dynamics (77). In contrast, miR-1 is involved in cell junctions (78) and has regulatory functions on cell proliferation and cell cycle progression (79, 80).

Among the DE novel miRNAs, we found three miRNAs (novel_miR_622, novel_miR_980, and novel_miR_64) targeting the DE genes that were related to reproduction and ovarian maturation, such as *irs1*, *adam12*, *hecw2*, and *dnm2* (61, 63, 67, 71). The tissue distribution results revealed that expression levels of these three novel miRNAs were low in gonads. These results were consistent with the observation that IGF3 downregulated the three miRNAs. IGF3 may induce maturation *via* these three miRNAs. However, the expression of the candidate circRNA (Lachesis_group5:6245955|6270787) differed between the ovary and testis (i.e., expression levels were low in the ovary and high in the testis). This circRNA may play a role in the reproduction of *S. argus*; however, it may bias towards male sex. The present results demonstrated that the ncRNAs may be involved in IGF3-induced ovarian maturation in *S. argus*. These results are expected to provide useful information about fish reproduction.

## Conclusions

In this study, circRNA and miRNA expression profiles involved in IGF3-induced ovarian maturation in *S. argus* were identified. An enrichment analysis of host genes of DE circRNAs and target genes of DE miRNAs revealed the roles of several pathways related to reproductive functions and oocyte maturation, such as the Hedgehog, PI3K-Akt, Apelin, Notch, insulin, and Rap1 signaling pathways. A circRNA–miRNA–mRNA regulatory network was constructed, from which DE genes involved in reproduction were identified, such as *ccnd2*, *hecw2*, *dnm2*, *irs1*, *adam12*, and *cdh13*. These DE genes are involved in oocyte maturation, such as oocyte meiosis and ECM remodeling. Based on the regulatory network and tissue distribution, we identified one circRNA (Lachesis_group5:6245955|6270787) and three miRNAs (novel_miR_622, novel_miR_980, and novel_miR_64) that may exert regulatory functions in IGF3-induced ovarian maturation in *S. argus*. Taken together, this study provided a novel insight into the molecular mechanisms underlying the effects of IGF3 in the ovary and highlighted the roles of circRNAs and miRNAs in *S. argus* reproduction.

## Data availability statement

The data presented in the study are deposited in the NCBI repository, accession number PRJNA849653.

## Ethics statement

The animal study was reviewed and approved by Animal Research and Ethics Committees of Fisheries College of Guangdong Ocean University.

## Author contributions

ZL: investigation, data curation, formal analysis, and writing—original draft. YG: investigation and methodology. CN: data curation and formal analysis. HDC: data curation and formal analysis. CH: methodology and resources. GZ: methodology. HH: data curation, and writing—review and editing. GL: methodology and resources. HPC: data curation, funding acquisition, methodology, resources, supervision, writing—original draft, and writing—review and editing. All authors contributed to the article and approved the submitted version.

## Funding

This article was supported by National Natural Science Foundation of China (Nos. 32273131 and 32172971), South China Aquaculture Breeding Funding (2022RHDKFKT01), the Key Research and Development Program of Guangdong (2021B202020002), the talent team tender grant of Zhanjiang Marine Equipment and Biology (2021E05035), the Guangdong Basic and Applied Basic Research Foundation (2019A1515010958), and the Southern Marine Science and Engineering Guangdong Laboratory (Zhanjiang) (ZJW-2019-06).

## Conflict of interest

Author HDC is employed by Guangdong Havwii Agriculture Group Co., Ltd, Zhanjiang, China. CH and GZ are employed by Hainan Chenhai Aquatic Co., Ltd, China.

The remaining authors declare that the research was conducted in the absence of any commercial or financial relationships that could be construed as a potential conflict of interest.

## Publisher’s note

All claims expressed in this article are solely those of the authors and do not necessarily represent those of their affiliated organizations, or those of the publisher, the editors and the reviewers. Any product that may be evaluated in this article, or claim that may be made by its manufacturer, is not guaranteed or endorsed by the publisher.
